# Thalamic structure and anastomosis in different hemispheres of moyamoya disease

**DOI:** 10.3389/fnins.2022.1058137

**Published:** 2023-01-09

**Authors:** Junwen Hu, Yongjie Wang, Yun Tong, Gaojun Lin, Yin Li, Jingyin Chen, Duo Xu, Lin Wang, Ruiliang Bai

**Affiliations:** ^1^Department of Neurosurgery, The Second Affiliated Hospital, Zhejiang University School of Medicine, Hangzhou, China; ^2^Clinical Research Center for Neurological Diseases of Zhejiang, Hangzhou, China; ^3^Affiliated Cixi Hospital of Wenzhou Medical University, Ningbo, China; ^4^Zhejiang University School of Medicine, Hangzhou, China; ^5^Department of Radiology, The Second Affiliated Hospital, Zhejiang University School of Medicine, Hangzhou, China; ^6^Key Laboratory of Biomedical Engineering of Ministry of Education, College of Biomedical Engineering and Instrument Science, Zhejiang University, Hangzhou, China; ^7^Department of Physical Medicine and Rehabilitation of the Affiliated Sir Run Run Shaw Hospital and Interdisciplinary Institute of Neuroscience and Technology, Zhejiang University School of Medicine, Hangzhou, China; ^8^MOE Frontier Science Center for Brain Science and Brain-Machine Integration, School of Brain Science and Brain Medicine, Zhejiang University, Hangzhou, China

**Keywords:** moyamoya disease, brain structure, cerebral angiography, anastomosis, thalamus

## Abstract

**Objective:**

The progression of the asymptomatic hemisphere of moyamoya disease (MMD) is largely unknown. In this study, we investigated the differences in subcortical gray matter structure and angiographic features between asymptomatic and symptomatic hemispheres in patients with MMD.

**Methods:**

We retrospectively reviewed patients with MMD in consecutive cases in our center. We compared subcortical gray matter volume and three types of collaterals (lenticulostriate anastomosis, thalamic anastomosis, and choroidal anastomosis) between symptomatic and asymptomatic hemispheres. Symptomatic hemispheres were classified as ischemic hemisphere (i-hemisphere) and hemorrhagic hemisphere (h-hemisphere). Asymptomatic hemispheres were classified as contralateral asymptomatic hemisphere of i-hemisphere (ai-hemisphere), contralateral asymptomatic hemisphere of h-hemisphere (ah-hemisphere), bilateral asymptomatic hemispheres in asymptomatic group (aa-hemisphere).

**Results:**

A total of 117 MMD patients were reviewed, and 49 of them met the inclusion criteria, with 98 hemispheres being analyzed. The thalamic volume was found to differ significantly between the i- and ai-hemispheres (*P* = 0.010), between the i- and ah-hemispheres (*P* = 0.004), as well as between the h- and ai-hemispheres (*P* = 0.002), between the h- and ah-hemispheres (*P* < 0.001). There was a higher incidence of thalamic anastomosis in the ai-hemispheres than i-hemispheres (31.3% vs. 6.3%, *P* = 0.070), and in the ah-hemispheres than h-hemispheres (29.6% vs. 11.1%, *P* = 0.088). Additionally, the hemispheres with thalamic anastomosis had a significantly greater volume than those without thalamic anastomosis (*P* = 0.024). Univariate and multivariate logistic regression analysis showed that thalamic volume was closely associated with thalamic anastomosis.

**Conclusion:**

The thalamic volume and the incidence of thalamic anastomosis increase in asymptomatic hemispheres and decrease in symptomatic hemispheres. Combining these two characteristics may be helpful in assessing the risk of stroke in the asymptomatic hemispheres of MMD as well as understanding the pathological evolution of the disease.

## Introduction

Moyamoya disease (MMD) is a type of chronic cerebrovascular disease characterized by progressive steno-occlusive changes at the terminal of the internal carotid arteries and the puff of smoke-like vascular network at the base of the brain (Suzuki and Takaku, 1969). Cerebral ischemia and intracranial hemorrhage are the two primary phenotypes of MMD. Despite the development of abnormal vessels on both sides of the brain, patients with MMD do not always have bilateral symptoms (Sun et al., 2022). Thus, there is a distinction between the symptomatic and asymptomatic hemispheres. Considering the potential for an asymptomatic hemisphere to become symptomatic as the disease advances (Kuroda et al., 2007), researchers are keen to understand the progression of the asymptomatic hemisphere (He et al., 2020; Lai et al., 2022).

“Moyamoya” vessels are the notably dilated perforating arteries in the basal ganglia and thalamus that conduct an important collateral circulation (Kuroda and AMORE Study Group, 2015). Abnormal circulation leads to structural changes in the brain. Lenticulostriate, thalamic, and choroidal anastomosis are the three primary collateral patterns of MMD, which have been described in detail in the symptomatic hemispheres (Funaki et al., 2018; Fujimura et al., 2019). However, the knowledge of the collateral circles in the asymptomatic hemisphere is still not known.

The aim of this study was to explore the differences of subcortical gray matter structure and angiographic features between asymptomatic and symptomatic hemispheres in patients with MMD. Clarifying the structural and angiographic characteristics of the asymptomatic hemisphere would be helpful to further understand the natural course of MMD and guide management of the asymptomatic hemispheres.

## Materials and methods

### Study design and cohort

We retrospectively reviewed consecutive patients diagnosed with MMD who visited our institution between July 2020 and May 2022. The diagnostic criteria were based on digital subtraction angiography (DSA) or magnetic resonance angiography (MRA) which were in accordance with the guideline (Clinical Guidelines Committee and Surgeons Branch of Chinese Medical Doctor Association, 2017). We enrolled adult patients with right-handed, no revascularization surgery before, and who had been free from ischemic/hemorrhagic attack for at least 1 month (Fujimura et al., 2019).

**FIGURE 1:**
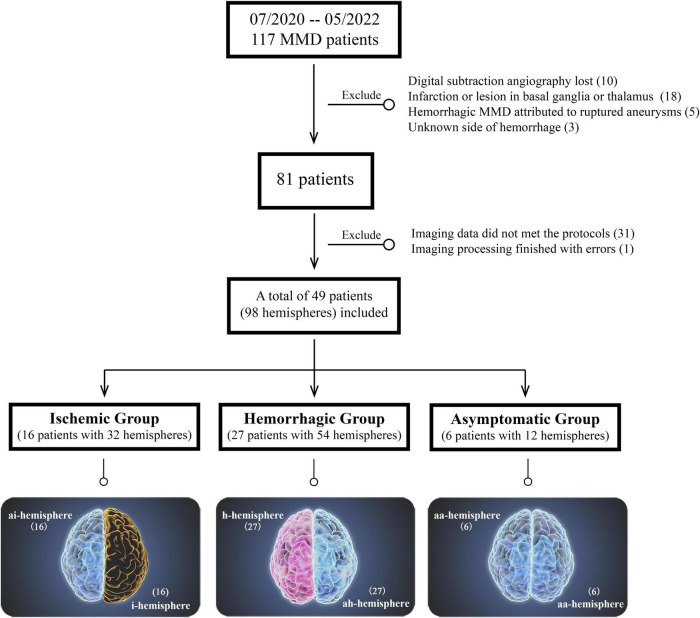
Patients’ inclusion and exclusion flowchart. The bottom is the classification of different hemispheres in moyamoya disease (MMD).

The exclusion criteria were as follows: (1) imaging data did not meet the imaging protocols or angiography lost; (2) hemorrhagic MMD attributed to ruptured intracranial aneurysms or which side of the brain suffering from hemorrhage could not be distinguished; (3) infarction or lesion in subcortical gray matter was excluded, which was considered to affect the structure of the subcortical region. The patients’ inclusion and exclusion flowchart was displayed in Figure 1. The Human Research Ethics Committee of the Second Affiliated Hospital of Zhejiang University approved this study (ID: 2020-064). Informed consent was obtained from every participant.

According to the clinical presentations and radiological findings, the included MMD patients were divided into ischemic group, hemorrhagic group, and asymptomatic group (without neurological symptoms or only with mild and unspecific symptoms such as headache or dizziness, and with negative findings on MR images). Their hemispheres were classified into five types proposed by Sun et al. (2022): ischemic hemisphere (i-hemisphere), hemorrhagic hemisphere (h-hemisphere), contralateral asymptomatic hemisphere of i-hemisphere (ai-hemisphere), contralateral asymptomatic hemisphere of h-hemisphere (ah-hemisphere), and bilateral asymptomatic hemispheres in asymptomatic group (aa-hemisphere) (Figure 1).

### Imaging protocol and processing

The MR imaging was completed on a 3T MR scanner (Discovery MR750, GE, Boston, MA, USA) with an 8-channel head coil. T1-weighted images (T1WI) were collected through 3D fast spoiled gradient-echo with repetition time (TR), 7.4 ms; echo time (TE), 3.1 ms; flip angle (FA), 8°; field of view (FOV), 256 mm × 256 mm; slices, 170; voxel size, 0.8 mm × 0.8 mm × 1.0 mm. T2-Flair images were acquired with TR, 8,400 ms; TE, 145.3 ms; FA, 90°; FOV, 512 mm × 512 mm; slices, 40; voxel size, 0.39 mm × 0.39 mm × 4.0 mm. Though it takes more scanning time for T1WI with high resolution, we get a more accurate structure of the subcortical gray matter. Time-of-flight MRA images were obtained by TR, 21 ms; TE, 2.5 ms; FA, 20°; FOV, 512 mm × 512 mm; slices, 129; voxel size, 0.39 mm × 0.39 mm × 1.4 mm.

Subcortical segmentation and volume calculation of T1WI were processed by FreeSurfer *v*6.0^1^ with the “recon-all” pipeline to automatically perform 31 processing steps for each subject, the technical details of these procedures were described in prior publications (Reuter et al., 2012). The volume of the subcortical gray matter (including basal ganglia, thalamus, and hippocampus) of each subject was then extracted. To control for the difference in brain size among subjects and the difference between the left and right hemispheres, the volume of basal ganglia, thalamus, and hippocampus were represented as a ratio to total subcortical gray matter volume.

### Collaterals evaluation

**FIGURE 2:**
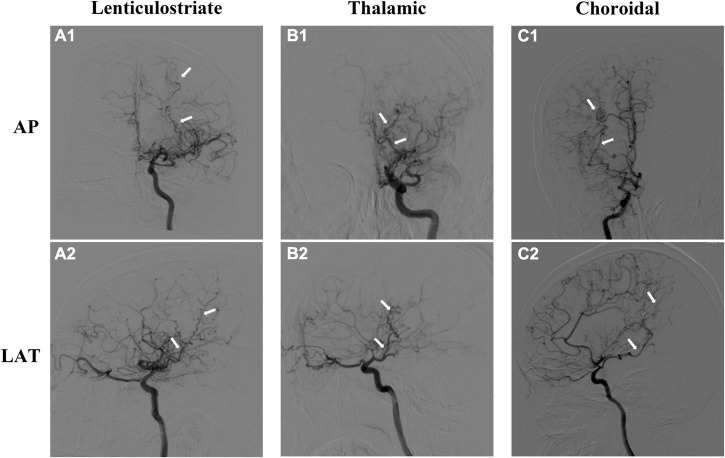
Three types of moyamoya disease (MMD) collaterals. The upper is the anteroposterior (AP) view of angiographic images, and the lower is the lateral (LAT) view. **(A1,A2)** Lenticulostriate anastomosis; **(B1,B2)** thalamic anastomosis; **(C1,C2)** choroidal anastomosis. The dilated and extended arteries are labeled with white arrows.

Three types of collaterals were classified according to previous research, which was named lenticulostriate anastomosis, thalamic anastomosis, and choroidal anastomosis, respectively (Funaki et al., 2018). Lenticulostriate anastomosis was defined as the anastomosis between the lenticulostriate artery and the cortex through the medial end of the medullary artery (meMedA) with at least 1 artery extending beyond the level of the pericallosal artery. Thalamic anastomosis was defined as the anastomosis between the thalamic perforator and the meMedA or the insular artery with at least 1 perforator extending beyond the position of the medial posterior choroidal artery (ChA). The last type was choroidal anastomosis, which was the anastomosis between the ChA and the meMedA with ChA deviated from the peripheral portion of the lateral ventricle (Figure 2; Funaki et al., 2018; Ge et al., 2020).

Each hemisphere of all patients was reviewed by two experienced neurosurgeons (Dr. Hu and Dr. Li) who were completely blinded to the patients’ clinical information through the anteroposterior and lateral views on DSA. If there was any difference, another senior neurosurgeon (Dr. Wang) was invited to discuss and reach a final consensus.

### Statistical analysis

Continuous variables were presented as the mean ± SD. The *t*-tests and Mann–Whitney *U* tests were used as appropriate when performed to determine group difference. The difference in the subcortical volume between groups was compared using the one-way ANOVA tests. The proportion of positive anastomosis and other categorical variables between groups were compared by the chi-square test. All variables from the univariate analysis using binary logistic regression with a *P* < 0.15 were further moved into a forward-stepwise multivariate analysis. Analyses were performed using SPSS *v*25 software (IBM, Armonk, NY, USA) and GraphPad Prism *v*8.0 (GraphPad Software, San Diego, CA, USA). Two-sided values of *P* < 0.05 was considered as statistically significant. All data generated and analyzed are available on reasonable request.

## Results

In this consecutive cohort of 117 patients with MMD, 10 patients had no documented DSA data; 18 patients had significant lesion in subcortical gray matter region; five patients had hemorrhage due to ruptured aneurysm; In three patients it was unable to distinguish the side of hemorrhage because of the equal volume of intraventricular hemorrhage in both sides; 31 patients did not met the imaging protocols of high-resolution T1WI; and 1 patient had an unknown error when performing image processing. The aforementioned patients were excluded from this study.

Finally, 49 patients with 98 hemispheres were enrolled. Among them, there were 16 patients in the ischemic group (including both 16 i- and ai-hemispheres) with symptoms including transient ischemic attack (*n* = 7, 43.8%), cerebral ischemia (*n* = 7, 43.8%), and paresthesia (*n* = 2, 12.5%). Twenty-seven patients were hemorrhagic MMD (including both 27 h- and ah-hemispheres) with the most frequent manifestation was primary intraventricular hemorrhage (IVH) (*n* = 9, 33.3%), followed by IVH with intracerebral hemorrhage (ICH) (*n* = 8, 29.6%), subarachnoid hemorrhage (SAH) (*n* = 3, 11.1%), IVH with SAH (*n* = 2, 7.4%), and ICH only (*n* = 5, 18.5%). And 6 cases in the asymptomatic group with 12 aa-hemispheres.

A total of 12 patients were ischemic MMD with symptoms including transient ischemic attack (*n* = 6, 27.3%), cerebral ischemia (*n* = 1, 4.5%), dizziness (*n* = 4, 18.2%), and headache (*n* = 1, 4.5%). A total of 10 patients were hemorrhagic MMD with the most frequent manifestation was primary IVH (*n* = 5, 22.7%), followed by IVH with ICH (*n* = 2, 9.1%), IVH with subarachnoid hemorrhage (*n* = 1, 4.5%), and ICH only (*n* = 2, 23.9%).

### Demographical and clinical characteristics

Table 1 summarizes the baseline characteristics of 49 patients enrolled in this study. The percentage of females was 85.7% (42/49) in total. There was no statistical difference in age, gender, body mass index (BMI), other personal/medical histories and Suzuki stage between the groups, except modified Rankin Scale (mRS) at admission (*P* = 0.006).

**TABLE 1:** Baseline characteristics of enrolled MMD patients.

	Ischemic group (16)	Hemorrhagic group (27)	Asymptomatic group (6)	*P*-value
Age (years)	45.9 ± 8.8	45.8 ± 7.5	46.3 ± 9.7	0.990
Gender (men/women)	2/14	5/22	0/6	0.488
BMI (kg/m^2^)	22.9 ± 2.3	23.1 ± 3.4	24.4 ± 4.1	0.577
Smoking (%)	2 (12.5%)	5 (18.5%)	0 (0%)	0.488
Alcohol (%)	1 (6.3%)	5 (18.5%)	0 (0%)	0.307
Hypertension (%)	6 (37.5%)	4 (18.2%)	1 (16.7%)	0.212
Diabetes mellitus (%)	1 (6.3%)	3 (11.1%)	2 (33.3%)	0.218
Hyperlipidemia (%)	5 (31.3%)	10 (45.5%)	1 (16.7%)	0.623
mRS at admission				0.006*
0	4	18	6	
1	10	5	0	
2	2	4	0	
Suzuki stage^#^				0.801
1	0	0	0	
2	2	5	2	
3	4	8	2	
4	7	9	2	
5	1	4	0	
6	2	1	0	

MMD, moyamoya disease; BMI, body mass index; mRS, modified Rankin Scale. *P < 0.01. ^#^The Suzuki stage was evaluated in both hemispheres and displayed the higher value here.

### Subcortical volume between different MMD hemispheres

**FIGURE 3:**
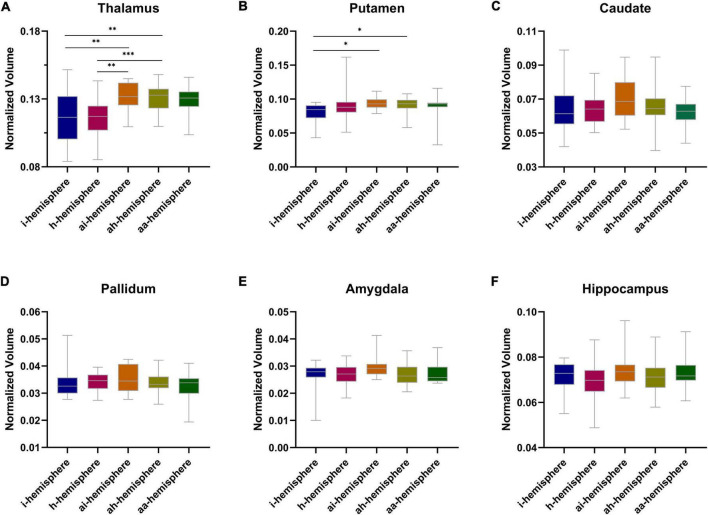
**(A–F)** Normalized subcortical gray matter volume between different hemispheres in moyamoya disease (MMD). *P < 0.05, **P < 0.01, ***P < 0.001.

A significant difference was noted in thalamic volume between the i- and ai-hemispheres (*P* = 0.010), the i- and ah-hemispheres (*P* = 0.004), the h- and ai-hemispheres (*P* = 0.002), as well as the h- and ah-hemispheres (*P* < 0.001) (Figure 3A). The putamen also displayed a significant difference between the i- and ai-hemispheres (*P* = 0.030), and the i- and ah-hemispheres (*P* = 0.034) (Figure 3B). As for other subcortical gray matter volumes, no statistical differences were found between the groups (Figures 3C–F). The total subcortical gray matter volume did not differ statistically between the different groups (Supplementary Figure 1).

### Collateral feature in different hemispheres

**FIGURE 4:**
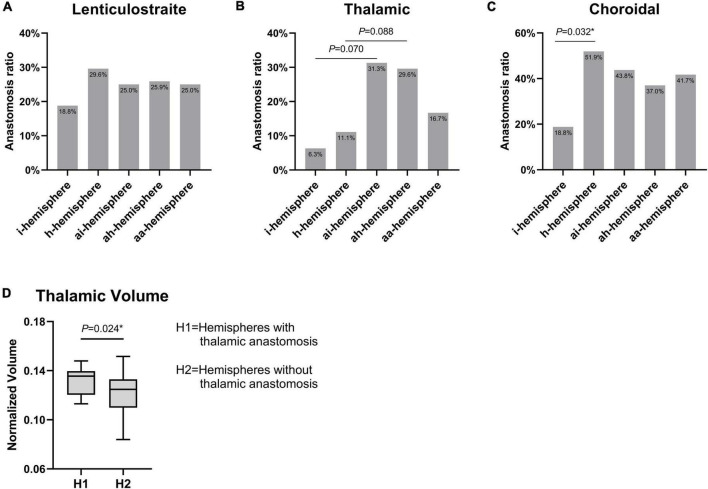
**(A–C)** Comparison of three types of collateral features among different hemispheres in moyamoya disease (MMD). **(D)** The difference of thalamic volume between hemispheres with thalamic anastomosis and those without thalamic anastomosis. *P < 0.05.

We observed lenticulostriate anastomosis was found in 24.9% of hemispheres, thalamic anastomosis in 19.0% of hemispheres, and choroidal anastomosis in 38.6% of hemispheres. There were no significant differences in the incidence of lenticulostriate anastomosis among the groups (Figure 4A). A difference toward statistical significance in the incidence of thalamic anastomosis was noted between the i- and ai-hemispheres (6.3% vs. 31.3%, *P* = 0.070), and between the h- and ah-hemispheres (11.1% vs. 29.6%, *P* = 0.088) (Figure 4B). The h-hemispheres had a significantly higher incidence of choroidal anastomosis than the i-hemispheres (18.8% vs. 51.9%, *P* = 0.032) (Figure 4C). Table 2 showed every *P*-value between the different hemispheres.

**TABLE 2:** Comparison of three types of collateral feature in different hemispheres.

	i-hemispheres	h-hemispheres	ai-hemispheres	ah-hemispheres
**Lenticulostriate anastomosis**				
i-hemispheres	/	/	/	/
h-hemispheres	0.340			
ai-hemispheres	0.500	0.515		
ah-hemispheres	0.442	0.500	0.621	
aa-hemispheres	0.521	0.544	0.672	0.640
**Thalamic anastomosis**				
i-hemispheres	/	/	/	/
h-hemispheres	0.521			
ai-hemispheres	0.070	0.101		
ah-hemispheres	0.069	0.088	0.587	
aa-hemispheres	0.389	0.494	0.334	0.332
**Choroidal anastomosis**				
i-hemispheres	/	/	/	/
h-hemispheres	0.032*			
ai-hemispheres	0.126	0.422		
ah-hemispheres	0.180	0.206	0.453	
aa-hemispheres	0.183	0.406	0.609	0.528

*P < 0.05.

### Higher thalamic volume in hemispheres with thalamic anastomosis

The thalamic volumes of the hemispheres with and without thalamic anastomosis was further compared. The results indicated that the hemispheres with thalamic anastomosis had significantly greater volume than those without this anastomosis (*P* = 0.024) (Figure 4D). In addition, the results of univariate logistic analysis found that gender (OR, 0.36; 95% CI: 0.11–1.24; *P* = 0.105), BMI (OR, 1.16; 95% CI: 0.98–1.36; *P* = 0.077) and thalamic volume (OR, 1.59; 95% CI: 1.05–2.42; *P* = 0.029) were associated with thalamic anastomosis. Furthermore, the multivariate logistic analysis indicated that thalamic volume (OR, 1.68; 95% CI: 1.07–2.63; *P* = 0.024) was the only factor that related to thalamic anastomosis (Table 3).

**TABLE 3:** Univariate and multivariate logistic regression analysis of factors associated with thalamic anastomosis.

Variables	Univariate		Multivariate	
	OR (95% CI)	*P*-value	OR (95% CI)	*P*-value
Age	0.98 (0.92–1.05)	0.586		
**Gender**	**0.36 (0.11–1.24)**	**0.105**	**2.84 (0.76–10.54)**	**0.120**
**BMI**	**1.16 (0.98–1.36)**	**0.077**	**1.18 (0.99–1.40)**	**0.061**
Smoking	0.54 (0.15–1.97)	0.353		
Alcohol	0.69 (0.17–2.82)	0.601		
Hypertension	0.77 (0.24–2.43)	0.653		
Diabetes mellitus	1.75 (0.20–15.14)	0.611		
Hyperlipidemia	1.45 (0.47–4.47)	0.513		
mRS at admission	0.82 (0.39–1.72)	0.593		
Suzuki stage	1.19 (0.79–1.79)	0.420		
**Thalamic volume**	**1.59 (1.05–2.42)**	0.029*	**1.68 (1.07–2.63)**	0.024*

OR, odds ratio; CI, confidence interval; BMI, body mass index; mRS, modified Rankin Scale. *P < 0.05.

The bold values indicate that three variables were analyzed in a univariate analysis with a *P* < 0.15 and are further analyzed in a multivariate analysis.

## Discussion

Identifying the brain volume, which is an important component of brain structure (Salinas et al., 2021), as well as angiographic features is an essential part of the research on MMD, as it can provide further information about the pathogenesis and neuropathology of the disease (Kazumata et al., 2019, 2020; Tahedl, 2020; Sun et al., 2022). However, previous studies have focused primarily on the symptomatic hemisphere. There has been no comparative study of the hemispheres with different characteristics, which is important for exploring the progression of MMD. Moreover, the proliferation of abnormal blood vessels in MMD initially develops at the skull base, which has a close relationship with the central core. Thus, we designed this study to examine the subcortical gray matter volume and collateral anastomosis in the different hemispheres of MMD, as well as the relationship between the two features.

Cerebral blood supply and hemodynamics are closely related to the brain structure (Zonneveld et al., 2015), decreased cerebral perfusion may contribute to brain atrophy (Alosco et al., 2013). Moon et al. (2020) demonstrated that subcortical gray matter volume decreases in patients with MMD using high-resolution imaging, but due to the small sample size and combined analyses of the symptomatic and asymptomatic hemispheres, the decreased volume had no statistical difference from healthy controls. Our study first examined subcortical gray matter volume between the different types of hemispheres in MMD and found that the thalamic volume in the symptomatic hemispheres (i- and h-hemispheres) was significantly smaller than that in contralateral asymptomatic hemispheres (ai- and ah-hemispheres).

We next investigated the angiographic feature between the hemispheres. The proportion of three different types of collateral anastomosis in this cohort was comparable to that of previous studies (Funaki et al., 2018; Fujimura et al., 2019). There was, for example, no difference between hemorrhagic and ischemic MMD in the incidence of lenticulostriate anastomosis, and hemorrhagic MMD had a significantly higher rate of choroidal anastomosis. Interestingly, the thalamic anastomosis rate in the symptomatic hemispheres (i- and h-hemispheres) was lower than that in the contralateral asymptomatic hemisphere (ai- and ah-hemispheres). Due to the limited sample size, the difference was not statistically significant, but the trend was evident.

Increased thalamic volume and the incidence of thalamic anastomosis in the asymptomatic hemisphere prompted us to explore the relationship between the two characteristics. Thalamic anastomosis arises from the thalamotuberal artery and thalamoperforating artery, which originate from the posterior communicating artery and posterior cerebral artery, respectively (Funaki et al., 2018). Both of these vessels are the primary blood supply of the thalamus (Bordes et al., 2020). The univariate and multivariate logistic analysis showed that there was a strong correlation between the thalamic volume and the incidence of thalamic anastomosis. Additionally, it has also been demonstrated in a previous study that increased cerebral blood flow corresponds to a larger brain volume (Erickson et al., 2011). Thus, we suggested that the thalamic volume increases as a result of the development of thalamic anastomosis.

The results of this study can be interpreted in two different ways. The first is that the hemispheres with a small thalamic volume and a lower incidence of thalamic anastomosis are more likely to suffer a stroke. The second assumption is that in the symptomatic hemispheres, thalamic anastomosis contracts after a stroke attack, which further causes atrophy of the thalamus. While in the ai- and ah-hemispheres, the thalamic volume and thalamic anastomosis are better preserved. Therefore, the combination of these two characteristics may provide insight into the risk of stroke in the asymptomatic hemispheres of MMD and the pathological development of the disease.

There are several limitations to be noted. Firstly, after taking into account certain factors that may influence the results of this study, the sample size appears to be small, which, in turn, limits the generalizability of the findings. A multicenter, large-sample study is needed to verify our results. Secondly, all of the patients included in this study were independent in their daily lives (scores between 0 and 2 on mRS), which might lead to a selection bias. Thirdly, Fujimura et al. (2019) have classified three types of anastomosis into two grades; however, it is unknown whether each grade has a different thalamic volume.

## Conclusion

In adult patients with MMD, the thalamic volume and the incidence of thalamic anastomosis increase in asymptomatic hemispheres and decrease in symptomatic hemispheres. Combining these two characteristics may be helpful in assessing the risk of stroke in the asymptomatic hemispheres of MMD as well as understanding the pathological evolution of the disease.

## Data availability statement

The raw data supporting the conclusions of this article will be made available by the authors, without undue reservation.

## Ethics statement

The studies involving human participants were reviewed and approved by the Human Research Ethics Committee of the Second Affiliated Hospital of Zhejiang University (ID: 2020-064). The patients/participants provided their written informed consent to participate in this study.

## Author contributions

JH and YW wrote the manuscript and collected the data. YT revised the manuscript. GL helped to evaluate statistical results. YL and JC assisted in writing the manuscript and contributed to the analysis of data. DX assisted in the MRI scan. JH, YL, and LW reviewed all imaging data. LW and RB conceived and designed the manuscript. All authors contributed to this article and approved the submitted version.

## Footnotes

1
http://surfer.nmr.mgh.harvard.edu
