# Supplementary material for: Thalamic structure and anastomosis in different hemispheres of moyamoya disease
Source: Front Neurosci. 2023 Jan 9;16:1058137. doi: 10.3389/fnins.2022.1058137 (PMC9869676; doi:10.3389/fnins.2022.1058137)
Supplement: Supplementary file 1 [file Data_Sheet_1.PDF]

Supplementary

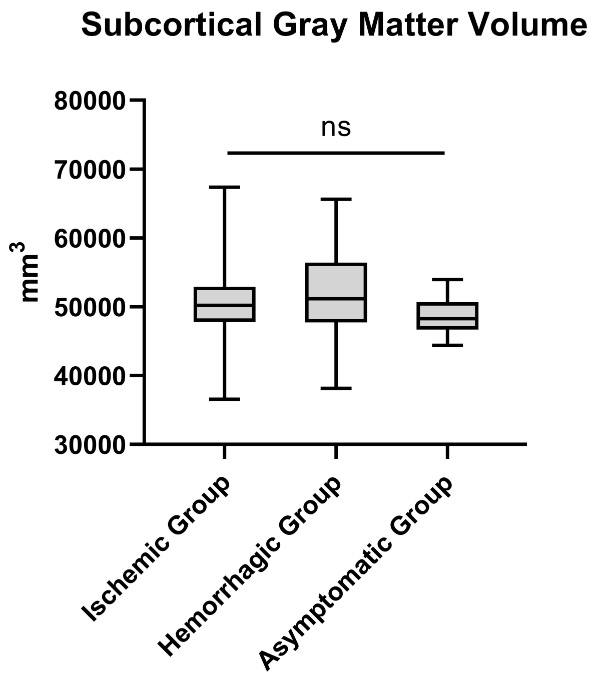

Supplementary Figure 1. The volume of subcortical gray matter in MMD does not differ statistically between the different groups.
